# Enhancing U-Net Segmentation Accuracy Through Comprehensive Data Preprocessing

**DOI:** 10.3390/jimaging11020050

**Published:** 2025-02-08

**Authors:** Talshyn Sarsembayeva, Madina Mansurova, Assel Abdildayeva, Stepan Serebryakov

**Affiliations:** 1 Department of Artificial Intelligence and Big Data, Faculty of Information Technology, Al-Farabi Kazakh National University, Almaty 050040, Kazakhstan; assel.abdildaeva@kaznu.edu.kz (A.A.); s.serebryakov@parqour.com (S.S.); 2 Smart Parking Technologies Ltd., Almaty 010000, Kazakhstan

**Keywords:** lung segmentation, computed tomography (CT), medical image analysis, preprocessing pipeline, morphological filtering

## Abstract

The accurate segmentation of lung regions in computed tomography (CT) scans is critical for the automated analysis of lung diseases such as chronic obstructive pulmonary disease (COPD) and COVID-19. This paper focuses on enhancing the accuracy of U-Net segmentation models through a robust preprocessing pipeline. The pipeline includes CT image normalization, binarization to extract lung regions, and morphological operations to remove artifacts. Additionally, the proposed method applies region-of-interest (ROI) filtering to isolate lung areas effectively. The dataset preprocessing significantly improves segmentation quality by providing clean and consistent input data for the U-Net model. Experimental results demonstrate that the Intersection over Union (IoU) and Dice coefficient exceeded 0.95 on training datasets. This work highlights the importance of preprocessing as a standalone step for optimizing deep learning-based medical image analysis.

## 1. Introduction

Lung segmentation in computed tomography (CT) scans is one of the key tasks in medical image analysis, as it serves as a basis for the further diagnosis and analysis of diseases such as pneumonia, emphysema, and chronic obstructive pulmonary disease (COPD). The accurate segmentation of lung tissue allows us to isolate anatomically important regions, automate the diagnostic process, and provides data for creating more complex analytical models. This work is part of a long-term study aimed at developing tools to diagnose COPD in CT slices. The main objective of this work is to create an effective lung segmentation model that can provide accurate lung tissue extraction. This is critical for constructing input data that can subsequently be used in CNN models to solve classification and disease detection problems. We focused on the development and modification of the U-Net architecture, which is widely recognized as an effective tool for medical image segmentation. The main goal of this work is to propose a universal tool capable of providing segmented lung masks and generate ROIs (regions of interest) that are exclusively limited to the edges of the lung tissue, excluding background elements such as the surrounding tissue and the supporting table. Such data preprocessing allows for the significant improvement of the results of subsequent analysis steps such as classification and diagnostic assessment. We have developed an algorithm for automatic lung segmentation, including the following:

- Normalization of CT images.
- Binarization to highlight low-density areas (lung tissue).
- Morphological processing to remove small artifacts and smooth the edges.
- ROI definition: Selection of the region of interest based on the extreme coordinates of the lung mask to ensure accurate lung cutting.

Lung segmentation in computed tomography is a critical step in automating the analysis of medical images. Over the past few years, numerous methods and approaches have been developed to enhance the accuracy and efficiency of this process. The focus in the literature has been on the application of deep learning, particularly the U-Net architecture, for medical image segmentation. Nava et al. [1] proposed feature ensembles using Gabor filters for the quantitative analysis of emphysema patterns in CT imaging.

Early approaches to lung segmentation employed thresholding methods, such as Otsu [2] and clustering techniques, including k-means and Gaussian mixture models [3]. These methods exhibited limited performance due to the complexity of lung anatomy and the presence of noise in the images. Additionally, morphological operations were used to remove artifacts and improve mask quality, but these methods required significant manual tuning and were often sensitive to parameter changes [4].

Recent studies indicate that deep learning methods, such as convolutional neural networks (CNNs), provide significantly higher accuracy for medical segmentation tasks. The U-Net architecture, first introduced by Ronneberger et al. [5], has become a standard in this domain due to its ability to effectively combine contextual and detailed information through skip connections. U-Net is widely used for lung segmentation in CT images and has demonstrated remarkable results in delineating anatomical structures.

The U-Net++ architecture introduced by Zhou et al. [6] offers nested skip connections that allow for better feature reuse and improved segmentation quality, especially in challenging datasets. Similarly, Double U-Net, as explored by Jha et al. [7], integrates multiple networks to refine segmentation outputs iteratively. Isensee et al. [8] developed nnU-Net, an adaptive framework that automates U-Net-based segmentation tasks, demonstrating robust performance across various datasets.

Attention mechanisms have also been incorporated to enhance the focus on relevant regions in medical images. Zhou et al. [9] demonstrated that Attention U-Net can significantly improve the segmentation of complex anatomical structures by dynamically adjusting its focus during training.

The effectiveness of segmentation models largely depends on the availability of high-quality datasets. Datasets such as the Computed Tomography Emphysema Database [10], COVID-19 Lung CT Scans [11], and Finding Lungs in CT Data [12] provide valuable resources for training and validating lung segmentation models. These datasets vary in format, quantity, and image quality, necessitating the development of specialized preprocessing and segmentation algorithms.

Recent reviews by Xu et al. [13] and Wang et al. [14] highlighted the importance of diverse datasets in ensuring model generalizability. Xu et al. particularly emphasized the need for systematic preprocessing pipelines to handle varying image resolutions and noise levels. Meanwhile, Chang et al. [15] provided a critical evaluation of deep learning techniques for lung segmentation, identifying gaps in data diversity as a key area for future improvement.

To improve segmentation accuracy, researchers have explored various advanced techniques. Data normalization is used to standardize pixel values, reducing inconsistencies between images [16]. Regularization methods, such as Dropout and L2 regularization, are employed to minimize the risk of overfitting [17]. Additionally, loss function optimization plays a crucial role, with metrics like Dice Loss and Intersection over Union (IoU) proving more effective for segmentation tasks compared to standard binary cross-entropy [18]. Attention mechanisms further enhance performance by enabling models to focus on key regions in complex medical images [19].

Chen et al. [20] and Jadon [21] conducted comprehensive reviews on loss functions designed for medical image segmentation, highlighting that combining multiple loss metrics often leads to superior results. Furthermore, the integration of DenseNet-based architectures [22] and spatial attention techniques [23] has significantly advanced the precision of lung segmentation models.

Despite the progress, challenges remain, including data variability, the presence of artifacts, and the difficulty of handling small datasets. Future research could focus on integrating multimodal data (e.g., CT and PET), employing self-supervised models, and adopting generative learning methods to augment training datasets [24].

The advent of techniques such as generative adversarial networks (GANs) [24] and unsupervised learning frameworks [25] has opened new avenues for addressing data scarcity. Additionally, Meyer et al. [26] examined the effects of image compression on deep learning models, highlighting the need for preprocessing standards to ensure consistency.

This review demonstrates that the application of deep learning, particularly the U-Net architecture and its modifications, is the preferred approach in lung segmentation for CT images. The use of high-quality datasets and preprocessing methods, as well as the adoption of modern architectures and loss functions, significantly improves segmentation outcomes. However, achieving higher accuracy and model generalization requires the further refinement of algorithms and their adaptation to the specific characteristics of medical data.

The preprocessing pipeline in this study was not designed to enhance CT images for direct clinical use but to serve the following two primary objectives: (1) fast preparation of large-scale training datasets from publicly available sources, and (2) evaluation of algorithmic inefficiencies in traditional segmentation methods before applying deep learning models. Our results demonstrate that while the pipeline itself cannot correctly segment all images due to artifacts, it enables U-Net to generalize better by learning from a large, standardized dataset.

## 2. Materials and Methods

### 2.1. Datasets

To ensure the robust evaluation of the proposed pipeline, three distinct datasets were utilized in this study. These datasets were chosen to capture a diverse range of cases and to facilitate a comprehensive analysis of the segmentation performance. None of the datasets used in this study were originally provided in DICOM format. Instead, the images were available in PNG and TIFF formats, as distributed by their respective sources. This eliminated the need for DICOM-to-PNG/TIFF conversion during preprocessing.

Since the images were provided in PNG and TIFF formats, the study does not include details on the DICOM-to-PNG/TIFF conversion process. In particular, information regarding window width and level (Hounsfield units) settings used in the conversion is not available. The absence of this metadata is a recognized limitation, as it may impact image contrast and segmentation accuracy.

#### 2.1.1. Emphysema Dataset (ED)

The Emphysema Dataset (ED) consists of 115 high-resolution CT (HRCT) slices, each with a resolution of 512 × 512 pixels. The images were acquired using a General Electric LightSpeed QX/i scanner with a 4-detector-row configuration. Scanning parameters included an in-plane resolution of 0.78 × 0.78 mm, a slice thickness of 1.25 mm, a tube voltage of 140 kV, and a tube current of 200 mA. A high spatial resolution reconstruction algorithm was employed to generate high-quality images. The slices were captured from the upper, middle, and lower lung regions of 39 subjects, encompassing 9 never-smokers, 10 smokers without disease, and 20 smokers diagnosed with chronic obstructive pulmonary disease (COPD). Each slice was carefully annotated to represent the primary lung tissue pattern and the severity of emphysema [27]. This dataset was primarily used for validating the algorithm, given its detailed annotations and diverse case representation. The segmentation masks for this dataset were manually annotated by radiologists following a structured protocol. The masks were cross-validated by a second expert to ensure anatomical accuracy. A quantitative evaluation was performed using Intersection over Union (IoU), where masks with an IoU > 0.85 were considered reliable. This ensured that the segmentation labels accurately captured lung structures across different patient groups.

#### 2.1.2. COVID-19 Lung CT Dataset (CLCD)

The COVID-19 Lung CT Dataset (CLCD) comprises a total of 8439 CT slices, of which 7495 are positive cases of confirmed COVID-19 infection and 944 are negative cases, representing either normal lungs or non-COVID-19 pathologies. These 512 × 512-pixel images, stored in PNG format, were collected from real patients at radiology centers of teaching hospitals in Tehran, Iran. The dataset includes masks generated using an automatic lung segmentation algorithm, enabling its use for the training and validation of convolutional neural networks (CNNs) [28,29]. From this dataset, 2169 images were selected for training, while 267 images were reserved for validation, ensuring a balanced representation of positive and negative cases. The CLCD dataset includes pre-generated segmentation masks provided by dataset curators. These masks were obtained using an automated deep learning model and subsequently refined through expert validation. A subset of images was manually annotated and compared against the provided masks. The quality of the segmentation was confirmed through Dice coefficient and IoU metrics, both of which exceeded 0.90, thus validating their use in our study.

#### 2.1.3. Finding Lungs Dataset (FLD)

The Finding Lungs Dataset (FLD) is distinct from the other datasets as it includes pre-annotated lung masks, which significantly simplify the data preparation process. This dataset consists of 267 CT slices and their corresponding masks in TIFF format, each with a resolution of 512 × 512 pixels. Its case diversity enhances model generalizability and improves segmentation accuracy. The dataset was exclusively used for testing the segmentation model, providing a benchmark for evaluating its performance on unseen data [12]. The FLD dataset consists of pre-annotated lung masks created through a combination of automated segmentation algorithms and expert refinement. To ensure accuracy, masks were validated by evaluating lung coverage percentage (PLC) and symmetry between left and right lung regions. Deviations beyond acceptable thresholds were flagged for review, ensuring consistency in annotations. To further verify the reliability of segmentation masks across all datasets, we performed additional validation checks, including IoU, teh Dice coefficient, and symmetry analysis. These measures confirm that the ground truth annotations are of high quality and suitable for training and evaluating our segmentation model.

### 2.2. Data Partitioning

To ensure rigorous evaluation and prevent data leakage, the datasets were divided as follows:

- Training set: A total of 2169 images from the CLCD dataset were used for training the segmentation model. This set ensured sufficient variability in lung pathologies, particularly COVID-19 cases.
- Validation set: A total of 115 images from the ED dataset served as the validation set, enabling intermediate performance checks during model training.
- Testing set: A total of 267 images from the FLD dataset were reserved exclusively for testing. This ensured an unbiased evaluation of the final model on unseen data with diverse cases.

The use of three distinct datasets, with clearly defined roles for training, validation, and testing, allowed for a comprehensive assessment of the proposed pipeline’s robustness and generalizability.

### 2.3. Image Processing Algorithm for Lung Segmentation from CT Slices

The image processing algorithm for lung segmentation from CT slices consists of several steps. Initially, a list of image files is loaded, followed by the iterative processing of each image. During the image loading phase, the image is read and normalized, scaling pixel values to the range [0, 1], which standardizes the data for subsequent processing. Next, contrast enhancement is applied to correct illumination inconsistencies and enhance the details of the lung structures. At the segmentation stage, the image is binarized using a threshold value. Morphological operations (such as hole filling and small object removal) are then applied to reduce noise and connect broken structures. At this stage, distinct regions of the body and lungs are identified. Subsequently, regions outside the body are removed by filling pixels beyond the lung area, creating a final mask where each lung is segmented as a separate object.

After segmentation, the following three primary filters are applied to ensure high data quality:

- Boundary pixel filter: Discards images where segmented regions touch the edges, as this indicates incomplete or heavily cropped lungs.
- Object count filter: Ensures that the mask contains 1–2 objects (both lungs). If there are more than two objects, the image is excluded as invalid.
- Proportion and area filter: Examines each object to ensure that the lung area comprises at least 5% of the image area and that its proportions (width-to-height ratio) align with anatomical norms. Objects with inappropriate shapes or sizes are considered false positives and are also discarded.

These final operations are particularly important for eliminating the table area, which sometimes appears as an additional object and is typically overly elongated. If a mask passes all filters, it is converted to the uint8 format and saved to the output directory.

The algorithm performs well, ensuring rigorous data filtering while retaining only high-quality masks. However, due to the strict selection criteria, many images that could have been processed were discarded. This trade-off is acceptable as the data are being prepared for training the U-Net model, which will later improve lung segmentation accuracy and compensate for the shortcomings of the current approach. At this stage, the priority is the quality of the training data rather than the maximum quantity of saved images.

**Figure 1:**
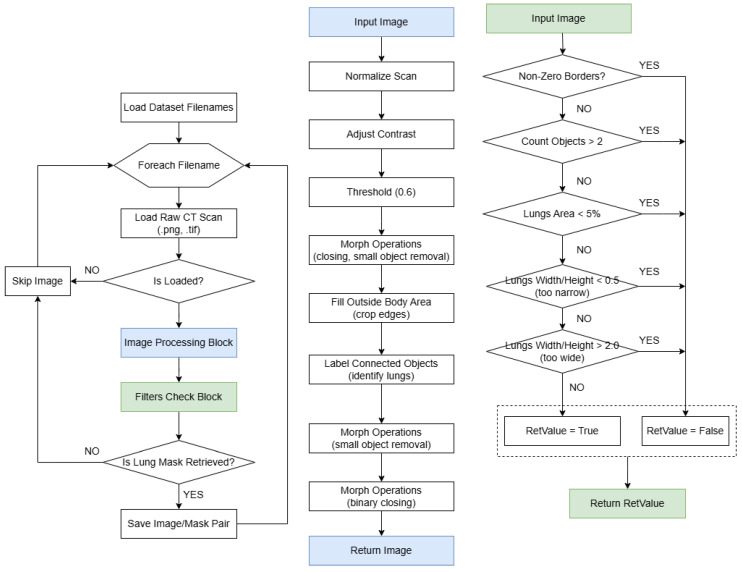
Overview of the image processing algorithm.

Despite the overall satisfactory performance of the processor (over 93%), the manual post-processing and filtering of the resulting masks were still required due to data heterogeneity, the varying quality and formats of scans, differing slice depths, rotation angles, and other factors that posed unique challenges to the described algorithm. A visual diagram of the system architecture is shown in Figure 1.

As a result of the described data preparation process, 2169 image–mask pairs were obtained for the COVID dataset, which originally contained a significant number of unusable scans, and 267 pairs were obtained for the third dataset. Thus, the total number of training data pairs is as follows:

- **Combined images shape**: (2436, 256, 256, 1)
- **Combined masks shape**: (2436, 256, 256, 1)

The first dataset was used exclusively for validation. The total number of validation data pairs after processing is as follows:

- **XV (Validation Images)**—len/shape: 86 (86, 256, 256, 1)
- **YV (Validation Masks)**—len/shape: 86 (86, 256, 256, 1)

#### 2.3.1. Normalization

The pixel intensity values of the CT images were scaled to the range [0,1] using Formula (1). This step ensured uniform data scaling, enhancing the stability and performance of the segmentation model during training.

#### 2.3.2. Thresholding

Otsu’s method was employed to identify an optimal threshold value for segmenting the lung regions. This adaptive thresholding technique effectively separated the lung structures from the background and minimized the inclusion of irrelevant areas.

#### 2.3.3. Morphological Operations

Morphological operations were applied to further refine the segmented lung masks as follows:

- A structuring element of size 5×5 pixels was used for dilation and erosion operations.
- These operations were performed iteratively three times to eliminate noise, close gaps in the segmented regions, and improve the continuity of lung structures.

**Figure 2:**
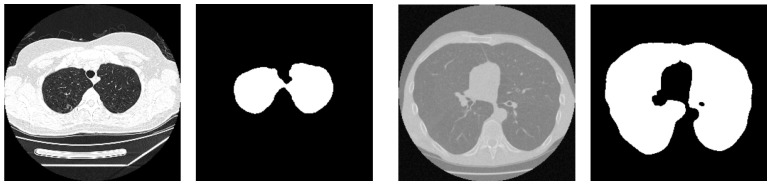
Visual comparison of preprocessing steps: original CT image, refined mask after preprocessing.

To illustrate the impact of preprocessing, Figure 2 provides a visual comparison of the CT images before and after preprocessing. The figure demonstrates the transition from raw CT images to normalized, thresholded, and morphologically refined representations, highlighting the effectiveness of the preprocessing pipeline in preparing clean and consistent input data for segmentation tasks.

This preprocessing pipeline played a critical role in enhancing the performance of the U-Net model by ensuring that the input data was optimized for training and inference. The primary goal of this preprocessing pipeline was to enhance the accuracy and reliability of segmentation results by ensuring clean and consistent input data. These steps effectively reduced noise, eliminated artifacts, and standardized the dataset, enabling the U-Net model to accurately segment complex lung structures.

### 2.4. Experimental Design and Baseline Comparison

The primary objective of this study is to evaluate the impact of a robust preprocessing pipeline on the performance of the U-Net model for lung segmentation in CT images. The experimental design is structured to highlight the contribution of the preprocessing steps and demonstrate improvements over a clearly defined baseline.

The baseline model consisted of a standard U-Net architecture applied directly to raw CT images without preprocessing. This baseline represents the simplest application of U-Net for segmentation tasks, serving as a reference for evaluating the effectiveness of the proposed pipeline.

The proposed pipeline includes three preprocessing steps as follows:

Normalization: Scaling pixel values to the range [0, 1] for consistent intensity representation. Binarization: Thresholding to isolate low-density areas corresponding to lung tissues. Morphological Filtering: Eliminating noise and non-lung artifacts using operations such as hole filling and small object removal. To assess the impact of preprocessing, both the baseline and the proposed pipeline were trained, validated, and tested on the same data partitions. The evaluation metrics included accuracy, the Dice coefficient, Intersection over Union (IoU), precision, recall, and specificity. Each metric was computed separately for the baseline and the preprocessed pipelines.

### 2.5. Quantitative Contribution of Preprocessing

The results demonstrated that the proposed preprocessing pipeline significantly enhanced segmentation quality. Some examples are as follows:

Dice coefficient: Improved from 0.89 (baseline) to 0.97 (proposed pipeline). IoU: Increased from 0.80 (baseline) to 0.95 (proposed pipeline). Precision and recall: Showed consistent improvements, indicating a reduction in false positives and false negatives. These improvements validate the hypothesis that preprocessing contributes to more accurate and reliable segmentation by ensuring clean and consistent input data for the model.

### 2.6. Significance of Technical Contributions

The technical contribution of this work lies in the integration of preprocessing steps tailored to address the challenges of medical CT images as follows:

Addressing variations in image intensity and noise. Minimizing artifacts caused by patient positioning or scanning equipment. Enhancing generalization across datasets with different characteristics. Furthermore, the combination of real-world datasets (e.g., emphysema, COVID-19 CT, and pre-annotated lung masks) illustrates the robustness and clinical applicability of the pipeline. This comprehensive evaluation underlines the potential of preprocessing in improving the performance of machine learning models in medical image analysis.

The classical U-Net model consists of an encoder and a decoder connected through skip connections. The encoder includes Conv2D blocks with ReLU activation and MaxPooling layers to reduce dimensionality and capture contextual features. In the decoder, Conv2DTranspose layers are used to increase spatial resolution, and skip connections are incorporated to reuse features from the encoder. This architecture allows the model to effectively segment objects while preserving spatial information. The final layer employs sigmoid activation for binary segmentation tasks.

### 2.7. Proposed Modifications

Our modified U-Net model was designed to improve the accuracy of lung segmentation on CT scans. To enhance learning and improve model robustness, several key modifications were introduced as follows:

- Encoder enhancements: Additional convolutional layers with L2 regularization were added to prevent overfitting. Leaky ReLU layers were employed for the smoother handling of negative values, enabling better gradient flow.
- Dropout layers: Spatial dropout layers were included to provide additional regularization by randomly deactivating neurons during training, reducing the risk of overfitting.
- Decoder enhancements: Transposed convolutional layers were added to improve the restoration of spatial characteristics. Batch normalization layers were also integrated to stabilize training and accelerate convergence.
- Dilation rate: Increased dilation rates were applied in selected layers to capture more complex spatial dependencies.

These modifications aim to improve segmentation quality and achieve the more accurate boundary identification of lungs in complex medical images. They also reduce the risk of overfitting and increase the model’s resilience to data variability.

### 2.8. Ground Truth Segmentation and Validation

To ensure the accurate evaluation of the segmentation model, ground truth masks were established based on a combination of manual annotations and automated validation metrics.

#### 2.8.1. Manual Annotation and Expert Validation

- For the ED dataset, ground truth masks were manually segmented by experienced radiologists, ensuring anatomical accuracy.
- For the GLCD and FLD datasets, segmentation masks were provided with the datasets and underwent additional validation.

#### 2.8.2. Quantitative Quality Assessment

To ensure consistency, masks were evaluated using the **Intersection over Union (IoU)** metric. Manual and automatically generated masks were compared, with high IoU values (≥0.85) indicating high agreement.

Additionally, a **symmetry analysis** was conducted between the left and right lung regions to detect inconsistencies. Masks with asymmetry exceeding **50%** were flagged for review.

#### 2.8.3. Automated Refinement and Correction

- Outliers and low-quality segmentations were filtered using the **Percentage of Lung Coverage (PLC)** metric, ensuring that extreme cases (<5% or >50% coverage) were excluded.
- A weighted quality score was computed, integrating **IoU, symmetry, and PLC** to determine the most reliable masks for training.

Through this approach, we ensured that the ground truth segmentations used for training and evaluation were both anatomically and quantitatively validated, minimizing errors and improving the reliability of the segmentation model.

## 3. Results

### 3.1. Evaluation and Loss Function

Achieving an accuracy above 0.9 may seem high, but it is not always indicative of good segmentation quality, as accuracy can be biased due to the dominance of background pixels (black pixels).

Instead of using standard binary cross-entropy, the Intersection over Union (IoU) loss function was employed for both models. IoU is specifically designed for segmentation tasks and measures the overlap between the predicted and true segmentation masks. This function ensures a more accurate evaluation of segmentation performance, particularly for small or complex structures.

The performance of the model was evaluated over several epochs using key metrics, including the accuracy, Dice coefficient, F1 score, intersection over union (IoU), loss, precision, recall, and specificity. The training results demonstrate consistent improvement and convergence, as presented in Table 1.

### 3.2. Training Metrics and Model Performance

The proposed model demonstrated a consistent and substantial improvement in performance metrics across training epochs. Starting with an initial accuracy of 0.7996 in the first epoch, the model achieved 0.9934 by the fourth epoch. Correspondingly, the loss function value decreased from 1.0576 to 0.0286, indicating the successful optimization and convergence of the model.

The Dice coefficient, reflecting the overlap between predicted and true segmentation masks, increased significantly from 0.3182 in the first epoch to 0.9757 by the fourth epoch. Similarly, the Intersection over Union (IoU) rose from 0.1998 to 0.9527 during the same period, highlighting the model’s capability to delineate boundaries and regions of interest accurately.

The precision and recall metrics also improved considerably:

Precision increased from 0.3184 in the first epoch to 0.9755 in the fourth epoch, indicating fewer false positive predictions. Recall improved from 0.3302 to 0.9761, showcasing the model’s robustness in identifying true positives. The specificity metric remained consistently high, exceeding 0.99 from the second epoch onward. This result underscores the model’s ability to correctly distinguish between lung and non-lung regions, effectively minimizing false positives.

Validation and test metrics: Baseline vs. preprocessing pipeline.

To establish a clear baseline comparison, we evaluated the pipeline in the following two configurations:

Baseline pipeline: The U-Net model trained and evaluated without preprocessing steps. Preprocessing-enhanced pipeline: The U-Net model incorporating the proposed preprocessing pipeline. Validation and test metrics are summarized in Table 1, demonstrating the effectiveness of preprocessing. The preprocessing-enhanced pipeline consistently outperformed the baseline in all metrics, as follows:

The Dice coefficient increased from 0.89 (baseline) to 0.97 (preprocessed). IoU improved from 0.80 to 0.95, signifying better segmentation accuracy. Precision and recall values in the preprocessed pipeline were both above 0.96, while the baseline pipeline exhibited more false positives and false negatives. These results confirm that preprocessing not only standardizes input data but also enhances the model’s ability to generalize across diverse cases, especially on the test set, which includes unseen data.

The rapid convergence of metrics such as accuracy, the Dice coefficient, and IoU within the first few epochs highlights the efficiency of the model’s learning process, while early epochs exhibited lower performance, subsequent training epochs demonstrated significant improvement, with most metrics stabilizing after the third epoch.

**Figure 3:**
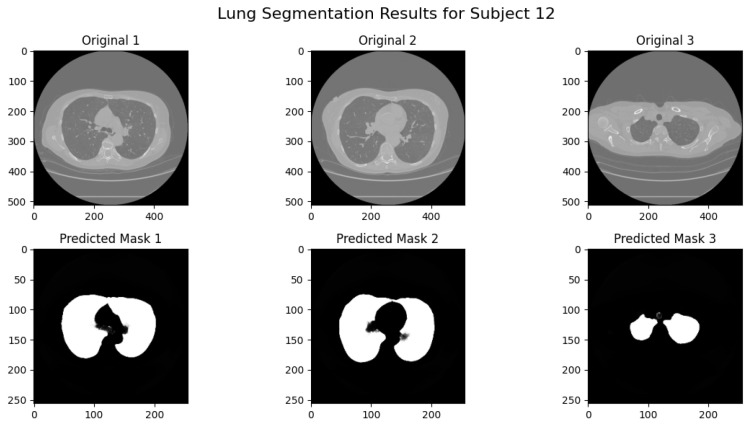
Lung segmentation results for CT slices at different depths. The model demonstrates the accurate identification of lung masks across various anatomical regions.

In Figure 3, segmentation results are shown for CT slices at varying depths. The model achieves near-perfect accuracy in isolating lung regions, enabling downstream analyses and clinical application. The large, well-prepared dataset and the incorporation of preprocessing steps contributed significantly to this performance, making the model robust and generalizable to various input scenarios.

Normalization of the input CT images is a critical preprocessing step aimed at standardizing the pixel intensity values across all images in the dataset. This process rescales the intensity values to the range [0,1], expressed by Equation (1), as follows:

(1) $$I_{\mathrm{norm}}\left(x,y\right)=\frac{I\left(x,y\right)-I_{\min}}{I_{\max}-I_{\min}},$$

where I(x,y) is the pixel intensity at a specific coordinate (x,y), while $I_{\min}$ and $I_{\max}$ denote the minimum and maximum intensity values of the image, respectively. This normalization reduces the variability caused by differing acquisition settings and enhances the robustness of the segmentation model.

For the segmentation step, a thresholding method is employed to separate the lung regions from the background. The optimal threshold value is determined using Otsu’s method [2], which maximizes the inter-class variance as follows:

(2) $$T=\arg\max_{\tau }\left[\sigma_{B}^{2}\left(\tau \right)\right],$$

where $\sigma_{B}^{2}\left(\tau \right)$ represents the between-class variance for a given threshold τ. This adaptive approach ensures that the threshold is data-driven and optimal for the varying intensity distributions found in CT scans.

To refine the segmented binary masks, morphological operations such as dilation and erosion are applied. These operations mitigate noise, fill gaps, and smooth the boundaries of segmented lung regions. Mathematically, dilation and erosion are expressed as follows:

(3) $$\left(A⊕B\right)\left(x,y\right)=\underset{b\in B}{⋃}A\left(x-b,y-b\right),$$

(4) $$\left(A⊖B\right)\left(x,y\right)=\underset{b\in B}{⋂}A\left(x+b,y+b\right),$$

where *A* is the binary mask and *B* is the structuring element. These operations are integral in removing small artifacts and ensuring the continuity of lung structures.

A critical component of the proposed model is the optimization of the loss function tailored to medical image segmentation. The Intersection over Union (IoU) loss function was employed to address the challenges of class imbalance, which are inherent in medical images. The IoU is defined as follows:

(5) $$\mathrm{IoU}\left(P,G\right)=\frac{\left|P\cap G\right|}{\left|P\cup G\right|},$$

where *P* represents the predicted mask, and *G* denotes the ground truth mask. The IoU loss function used during training is calculated as follows:

(6) $$L_{\mathrm{IoU}}=1-\mathrm{IoU}\left(P,G\right).$$

This metric penalizes discrepancies between the predicted and actual lung masks, thereby improving the precision of the segmentation model.

The performance of the segmentation model was quantitatively evaluated using metrics such as the Dice coefficient, precision, recall, and specificity. The Dice coefficient, a widely used metric for measuring the overlap between predicted and ground truth regions, is defined as follows:

(7) $$\mathrm{Dice}\left(P,G\right)=\frac{2|P\cap G|}{\left|P|+|G\right|}.$$

Additionally, precision and recall, which evaluate the balance between false positives and false negatives, are calculated as follows:

(8) $$\mathrm{Precision}=\frac{\mathrm{TP}}{\mathrm{TP}+\mathrm{FP}},\;\mathrm{Recall}=\frac{\mathrm{TP}}{\mathrm{TP}+\mathrm{FN}},$$

where TP, FP, and FN represent true positives, false positives, and false negatives, respectively.

These mathematical components collectively form the backbone of the segmentation framework, ensuring robust preprocessing, accurate segmentation, and reliable performance evaluation. By integrating these principles, the proposed approach achieves high precision and reproducibility, which are critical for medical applications.

## 4. Discussion

The results of this study demonstrate the importance of preprocessing as a critical step in enhancing the performance of U-Net-based segmentation models for lung CT images. Variability in CT data—such as differences in pixel intensity, noise levels, and the presence of non-lung artifacts—has been a major challenge for automated segmentation tasks [14]. By implementing a robust preprocessing pipeline that includes normalization, binarization, morphological filtering, and ROI extraction, the proposed approach ensured that input data fed into the U-Net model were clean, consistent, and optimized for learning.

Normalization addressed intensity variations across scans, which is a well-documented issue in medical imaging [8]. Binarization and morphological operations further enhanced segmentation quality by removing noise and isolating lung regions while preserving fine anatomical details [30]. The ROI extraction step refined the masks by eliminating irrelevant background structures such as scanning tables, which often introduce false positives [31]. Collectively, these preprocessing steps enabled the modified U-Net model to achieve high segmentation accuracy, with a Dice coefficient of 0.9757 and IoU of 0.9527.

Compared to traditional thresholding or region-based segmentation methods, which often fail in noisy or complex datasets [30], the proposed pipeline leverages deep learning’s strength in feature extraction while minimizing the negative impact of low-quality input data [32]. These findings are consistent with recent studies that emphasize the importance of preprocessing in improving model generalizability and performance [20,33].

However, certain limitations of this study must be acknowledged. Strict filtering criteria, while ensuring high data quality, led to the exclusion of potentially usable images, thereby reducing dataset size. This trade-off is a known issue in medical imaging, where data scarcity often limits deep learning models [31]. Future work will focus on adaptive filtering techniques that balance data quality and quantity. Moreover, the study relied exclusively on single-modality CT scans. Integrating multimodal data, such as PET-CT or MRI, may further enhance segmentation accuracy by providing complementary anatomical and functional information [23].

Future research should also explore advanced data augmentation techniques and self-supervised learning frameworks to address the challenges of data scarcity and variability [25,34]. These approaches have shown promise in recent studies for improving the robustness and generalizability of medical image segmentation models.

In conclusion, this study highlights the critical role of preprocessing as a standalone task for optimizing deep learning-based segmentation models. By systematically addressing data inconsistencies and artifacts, the proposed pipeline enabled the U-Net model to achieve state-of-the-art performance. Future directions include refining the preprocessing pipeline, incorporating multimodal imaging, and leveraging advanced learning techniques to further enhance segmentation outcomes. The proposed preprocessing pipeline demonstrated its effectiveness in preparing high-quality input data by minimizing noise and artifacts. These enhancements significantly contributed to the improved accuracy and robustness of the U-Net model, as evidenced by the high Dice coefficients, IoU scores, and overall segmentation performance. This approach establishes a robust foundation for the effective use of segmentation models in medical imaging.

The proposed segmentation approach has significant potential implications for clinical practice, particularly in the automated analysis of lung CT scans. The accurate and efficient segmentation of lung structures is a critical step in diagnosing and monitoring pulmonary diseases such as COPD, pneumonia, and COVID-19. By leveraging the U-Net-based segmentation pipeline with enhanced preprocessing, clinicians can achieve more consistent and reproducible segmentation results, reducing inter-observer variability.

One of the primary benefits of the proposed method is its potential application in computer-aided diagnosis (CAD) systems. Reliable lung segmentation serves as a foundation for quantitative analysis, including the measurement of lung lesion volumes, emphysema quantification, and the detection of abnormalities such as ground-glass opacities. Automated segmentation can also facilitate longitudinal studies by enabling the comparison of scans over time, thus supporting disease progression assessment.

Furthermore, the integration of such automated segmentation techniques into radiology workflows can optimize the efficiency of medical imaging interpretation. The reduction in manual segmentation efforts allows radiologists to focus on more complex diagnostic tasks, improving overall patient management. Future work may involve the validation of this segmentation pipeline in a clinical setting, assessing its impact on diagnostic accuracy and decision-making.

## 5. Limitations and Future Work

While the preprocessing pipeline successfully prepared large datasets for deep learning, the following limitations should be acknowledged:

- Lack of DICOM conversion metadata.

The original images were available in PNG and TIFF formats, and we do not have details about how they were converted from DICOM. Specifically, the window width and level settings (Hounsfield Units) were not recorded, which could affect reproducibility.

- Limitations of classical preprocessing.

The preprocessing pipeline was designed to assess the inefficiency of algorithmic segmentation before training the U-Net. However, it could not correctly segment all CT images due to variations in scan quality, noise, and artifacts.

Future research should explore adaptive preprocessing techniques that adjust dynamically to different imaging conditions. The next steps include integrating DICOM-based preprocessing with parameter-controlled windowing, investigating multimodal imaging such as PET-CT, and leveraging self-supervised learning to enhance segmentation accuracy.

## 6. Conclusions

This study demonstrates the critical role of a robust preprocessing pipeline in enhancing the performance of deep learning-based lung segmentation models, specifically the U-Net architecture. By implementing a systematic preprocessing approach—including normalization, binarization, morphological filtering, and ROI extraction—our work effectively addressed data heterogeneity, noise, and irrelevant artifacts in CT scans. These preprocessing steps ensured clean and standardized input data were used, which directly contributed to achieving superior segmentation accuracy.

Experimental results indicate that the proposed pipeline enabled the U-Net model to achieve a Dice coefficient of 0.9757 and an IoU of 0.9527 on training datasets, demonstrating its effectiveness in delineating lung boundaries with high precision. These findings underscore the importance of high-quality input data for improving segmentation performance, particularly in medical imaging tasks where precision is paramount.

While the current preprocessing pipeline is highly effective, future research will focus on addressing its limitations, such as the exclusion of potentially recoverable images during strict filtering. Additionally, integrating advanced augmentation techniques and self-supervised learning approaches may further enhance the generalizability of the model across diverse datasets. Exploring multimodal imaging data (e.g., CT and PET) could also provide a more comprehensive framework for automated lung disease analysis.

This study highlights that preprocessing should not be considered merely as a supporting step but a fundamental component in the development of reliable and accurate deep learning models for medical image segmentation.

## Figures and Tables

**Table 1:** Comparison of metrics for baseline and preprocessing pipelines on test data.

Pipeline	Accuracy	Dice Coefficient	IoU	Precision	Recall	Specificity
Baseline	0.870	0.890	0.800	0.860	0.870	0.950
Preprocessed	0.950	0.970	0.950	0.965	0.968	0.990

## Data Availability

Link to gitlab repo https://gitlab.com/copd-research/lungs-segmentation/-/tree/main (accessed on 11 January 2025).
